# Quality of Care and Survival Outcomes Among Patients With Clinically Localized Prostate Cancer in Nigeria

**DOI:** 10.1200/GO-24-00504

**Published:** 2025-03-24

**Authors:** Musliu Adetola Tolani, Christian Agbo Agbo, Alan Paciorek, Shehu Salihu Umar, Rufus Wale Ojewola, Faruk Mohammed, Ernie Kaninjing, Muhammed Ahmed, Rebecca DeBoer

**Affiliations:** ^1^ Ahmadu Bello University Teaching Hospital, Ahmadu Bello University, Zaria, Nigeria; ^2^ Benue State University Teaching Hospital, Makurdi, Nigeria; ^3^ University of California, San Francisco, CA; ^4^ Lagos University Teaching Hospital, University of Lagos, Idi-Araba, Nigeria; ^5^ Georgia College and State University, Milledgeville, GA

## Abstract

**PURPOSE:**

Optimal survival outcomes of prostate cancer are best achieved through high-quality care for curable disease. In Nigeria, various barriers may impede the curative treatment of prostate cancer, yet their impact on care and patient outcomes remains anecdotal. This study assessed treatment quality, survival outcomes, and interhospital differences of these metrics among patients with clinically localized prostate cancer in Nigeria.

**METHODS:**

A retrospective study of patients with clinical stage T1-T3a, M0 prostate cancer at three tertiary hospitals in Nigeria over a 3-year period was conducted. Data on hospital sites, sociodemographics, clinicopathologic characteristics, quality metrics, imaging used, treatment, and survival status were collected. The primary end point was time from diagnosis to first treatment. Secondary end points were time from presentation to diagnosis, other prostate cancer quality metrics, all-cause survival, and interhospital differences in these metrics. Quality of diagnostics, treatments, and other outcomes were described and compared using Cox regression.

**RESULTS:**

This study included 110 patients with a median age of 67 years. Most (n = 66, 61%) had high-risk disease. The median time from tertiary hospital presentation to diagnosis was 31 days. Median time from diagnosis to first treatment of any type was 68 days, with radical radiotherapy was 117 days, and with radical prostatectomy was 104 days. Eighteen percent (n = 20) had guideline-concordant imaging for tumor staging, 67 patients (61%) received any treatment or active surveillance, and retention in care was 42%. Three-year all-cause survival was 41%. There was a significant difference in most quality metrics including guideline-concordant imaging and treatment across the hospital sites.

**CONCLUSION:**

Time to treatment was delayed beyond international benchmarks; quality of staging, treatment, and care process were suboptimal; and survival was poor amid geographical disparities in care.

## INTRODUCTION

Prostate cancer is the second most commonly diagnosed malignancy in men globally.^1^ Major disparities exist in the health care system infrastructure required for diagnosis and treatment of prostate cancer in low- and middle-income countries (LMICs) compared with high-income countries.^2-4^ Poor access to diagnostic facilities and specialist management are common barriers in LMICs. The delivery of definitive treatment, such as radical prostatectomy or radiotherapy, is also a challenge, with only 17.5% of those with localized disease receiving curative treatment in sub-Saharan Africa.^5^ In Nigeria, there are currently around 270 urologists and 63 radiation or clinical oncologists serving a population of 225 million people^6^ and only nine teletherapy and eight brachytherapy machines in government-funded centers.^7^ In addition, fragmentation of care results in patient attrition during follow-up.^8^ These health system gaps occur amid a dynamic background of cultural and personal beliefs, socioeconomic problems, and past hospital experiences, which affect the health-seeking behavior of patients.^9^

**CONTEXT**

**Key Objective**
What is the quality of care and survival rate of patients with clinically localized prostate cancer in Nigeria, and are there interhospital differences in these metrics?
**Knowledge Generated**
The cohort analysis demonstrated delays in treatment initiation; low concordance to imaging and treatment guidelines; and poor patient survival outcomes in Nigeria. Findings also suggest poor retention in care, and inequity in the socioeconomic status, clinical characteristics, and treatment quality of patients with prostate cancer living across Nigeria.
**Relevance**
These gaps should be the target for evidence-based interventions centered on service integration in the nonspecialist setting, social needs screening, geriatric evaluation, patient navigation, and guideline adoption to expand access and improve the delivery of prostate cancer care in Nigeria.

Optimal survival of prostate cancer is best achieved through early detection and high-quality curative treatment. In Nigeria, evidence to describe clinically relevant indicators of care quality, such as time to diagnosis and treatment, and patients' survival outcomes, is lacking. These indicators are important markers of both quality and equity in the management of clinically localized prostate cancer (T1-T3a, M0). Moreover, while public awareness has improved, interventions to reduce delays in clinical management, as recommended by the WHO, are largely still missing.^10^ Therefore, there is a need to characterize clinical delays, quality, and outcomes to identify specific targets for intervention. This study aimed to assess the time from first cancer-related tertiary hospital presentation to prostate biopsy, the time from diagnosis to first cancer-directed treatment, other quality metrics, survival outcomes, and interhospital differences in these metrics among patients with clinically localized prostate cancer in Nigeria.

## METHODS

### Study Design and Setting

This was a retrospective cohort study carried out at three public tertiary hospitals, one each in the North West (Zaria), North Central (Lafia), and South West (Lagos) geopolitical zones of Nigeria (see Appendix Table A1 for further background). Ethical approvals were obtained. Informed consent was waived because of the retrospective nature of the study.

### Study Population and Selection Criteria

Men diagnosed with clinically localized prostate cancer (T1-T3a, M0) from January 2018 to December 2020 at the three hospital sites were included, while those with metastatic disease were excluded.

### Sampling Power

One hundred ten patients were sufficient to detect a medium effect of a potential predictor on time to death with a hazard ratio (HR) of 2.0 and a minimum cumulative incidence over the duration of the study of 45% in the control group using a two-sided log-rank test with 5% type 1 error rate and 80% power.

### Data Collection

Patient information was extracted from hospital records and entered into data collection forms in OnaData, a secure web-based database system. It included the hospital site, sociodemographic and clinical characteristics, imaging used, and the type of definitive treatment received. Data on dates and documentation pertaining to prostate cancer quality metrics, date of first biochemical recurrence, and vital status were also collected (Appendix Table A2).

### Study End Points

The primary end point was time to treatment. Secondary end points were time to diagnosis; sociodemographic and clinical characteristics; concordance to the standard of care (Appendix Table A3) and other established prostate cancer quality metrics^11-14^; patient survival (biochemical recurrence-free survival and all-cause survival); and interhospital differences in these metrics.

### Data Analysis

Data were analyzed using Stata software, version 18.0 (StataCorp LLC, College Station, TX; Appendix Table A2 footnote). A *P* value of <.05 was considered significant.

## RESULTS

This study included 110 patients with a median age at diagnosis of 67 years. There were significant differences in educational status, occupational status, and site of previous care across the three hospital sites as shown in Table 1.

**TABLE 1:** Sociodemographic Characteristics of Patients With Prostate Cancer

Characteristic^a^	Zaria	Lagos	Lafia	Total	*P* ^b^
	42 (38)	39 (35)	29 (26)	110 (100)	
Age at diagnosis, years					
49-59, No. (%)	4 (10)	9 (23)	6 (21)	19 (17)	.474
60-69, No. (%)	19 (45)	16 (41)	8 (28)	43 (39)	
70-79, No. (%)	16 (38)	10 (26)	12 (41)	38 (35)	
80-95, No. (%)	3 (7)	4 (10)	3 (10)	10 (9)	
Median (IQR)	67 (64-74)	64 (60-75)	70 (61-75)	67 (61-75)	.684
Mean (SD)	68 (8)	67 (10)	67 (9)	67 (9)	
Marital status, No. (%)					
Married	41 (100)	27 (75)	25 (86)	93 (88)	.018
Widowed	0	8 (22)	4 (14)	12 (11)	
Divorced/single	0	1 (3)	0	1 (1)	
Missing data	1	3	0	4	
Educational status, No. (%)					
Nonformal	1 (8)	0	0	1 (2)	.007
Primary	0	1 (4)	6 (22)	7 (11)	
Secondary	0	6 (22)	9 (33)	15 (23)	
Tertiary	11 (92)	20 (74)	12 (44)	43 (65)	
Missing data	30	12	2	44	
Occupational status, No. (%)					
Class I (high)	9 (23)	7 (23)	2 (8)	18 (19)	.001
Class II (intermediate)	8 (21)	7 (23)	3 (12)	18 (19)	
Class III (low)	6 (15)	2 (7)	12 (46)	20 (21)	
Retired	16 (41)	8 (27)	8 (31)	32 (34)	
No occupation	0	6 (20)	1 (4)	7 (7)	
Missing data	3	9	3	15	
Insurance status, No. (%)					
No	37 (88)	15 (75)	28 (97)	80 (88)	.075
Yes	5 (12)	5 (25)	1 (3)	11 (12)	
Missing data	0	19	0	19	
Receiving care for another condition, No. (%)					
No	33 (82)	16 (62)	13 (45)	62 (65)	.005
Yes	7 (18)	10 (38)	16 (55)	33 (35)	
Missing data	2	13	0	15	

NOTE. Another condition represents patients receiving care for another condition in the same hospital before diagnosis.

Abbreviation: SD, standard deviation.

^a^ Percentages and association tests were computed for variables with known values.

^b^ *P* value from Pearson chi-squared test or Kruskal-Wallis test.

### Time to Treatment

The overall median time from diagnosis to the first cancer-directed treatment or active surveillance decision was 68 days (IQR, 51-120 days). The median time from diagnosis to first treatment with radical radiotherapy was 117 days (IQR, 80-152), radical prostatectomy was 104 days (IQR, 0-134), androgen-deprivation therapy (ADT) was 68 days (IQR, 51-165), and for active surveillance decision was 57 days (IQR, 50-63). The stratification of time to treatment by clinical tumor (cT) stage and risk group is shown in Appendix Table A4. Seven percent of all patients (n = 8) had their first treatment within 30 days of diagnosis, 38% (n = 42) were first treated within 90 days, and 39% (n = 43) never received any treatment (Appendix Table A5). A shorter time to treatment was observed in Lagos (HR, 2.37 [95% CI, 1.23 to 4.57]) and Lafia (HR, 2.51 [95% CI, 1.16 to 5.44]) compared with Zaria after controlling for D'Amico risk (*P* = .057) and age (*P* = .027). There was no statistically significant association with other patient or disease characteristics and imaging or treatment guideline status. Insured patients were more likely than uninsured patients to receive surgery instead of no treatment (relative risk, 10.9 [95% CI, 2.2 to 53.1]).

### Time to Diagnosis

The median time from presentation to diagnosis was 31 days (IQR, 13-50). Time to diagnosis was not associated with hospital site, socioeconomic or demographic status, clinical characteristics, and other quality metrics (data not shown).

### Quality Metrics

Less than half of the patients had documentation of family history (n = 52; 47%), at least 10 prostate biopsy cores obtained (n = 47; 46%), or cT stage recorded within 1 month of diagnosis (n = 46; 42%). There were significant differences in most of the quality metrics across sites (Table 2).

**TABLE 2:** Quality Metrics of Prostate Cancer Management in Nigeria

Characteristic	Zaria	Lagos	Lafia	Total	*P* ^a^
	42 (38)	39 (35)	29 (26)	110 (100)	
Documentation of family history, No. (%)					
No	41 (98)	9 (23)	8 (28)	58 (53)	<.001
Yes	1 (2)	30 (77)	21 (72)	52 (47)	
Documentation of PSA at diagnosis, No. (%)					
No	0	0	2 (7)	2 (2)	.058
Yes	42 (100)	39 (100)	27 (93)	108 (98)	
Prostate biopsy cores, No. (%)					
<10	29 (81)	0	27 (96)	56 (54)	<.001
≥10	7 (19)	39 (100)	1 (4)	47 (46)	
Documentation of Gleason's grade, No. (%)					
No	1 (2)	0	2 (7)	3 (3)	.222
Yes	41 (98)	39 (100)	27 (93)	107 (97)	
Documentation of cT stage within 1 month of diagnosis, No. (%)					
No	18 (43)	35 (90)	11 (38)	64 (58)	<.001
Yes	24 (57)	4 (10)	18 (62)	46 (42)	
Guideline-concordant treatment, No. (%)					
No	41 (98)	32 (84)	28 (97)	101 (93)	.046
Yes	1 (2)	6 (16)	1 (3)	8 (7)	
Concordant treatment for low risk, No. (%)					
No	1 (100)	3 (60)	3 (100)	7 (78)	.358
Yes	0	2 (40)	0	2 (22)	
Concordant treatment for intermediate risk, No. (%)					
No	3 (100)	15 (100)	16 (100)	34 (100)	NA
Yes	0	0	0	0	
Concordant treatment for high risk, No. (%)					
No	37 (97)	14 (78)	9 (90)	60 (91)	.058
Yes	1 (3)	4 (22)	1 (10)	6 (9)	
Retention at end of follow-up,^b^ No. (%)					
Retained in care	4 (10)	32 (82)	11 (38)	47 (43)	<.001
Lost to follow-up	37 (90)	7 (18)	18 (62)	62 (57)	
Missing data	1	0	0	1	
Patients with at least two follow-up hospital visits during the first year after treatment,^c^ No. (%)					
No	34 (81)	3 (8)	19 (66)	56 (51)	<.001
Yes	8 (19)	36 (92)	10 (34)	54 (49)	
Days to diagnosis					
Median (IQR)	25 (12-48)	37 (27-48)	29 (9-52)	31 (13-50)	.178
Count of patients, No.	37	39	27	103	
Days to any first treatment					
Median (IQR)	69 (45-190)	80 (55-120)	66 (46-97)	68 (51-120)	.539
Count of patients, No.	18	31	18	67	

Abbreviations: cT, clinical tumor; NA, not applicable; PSA, prostate-specific antigen.

^a^ *P* value from Pearson chi-squared test or Kruskal-Wallis test.

^b^ Percentages and association tests were computed for variables with known values.

^c^ PSA dates were used to compute optimal follow-up of patients within 1 year of diagnosis.

Table 3 shows that few patients at any hospital had complete tumor or metastatic evaluation. For evaluation of T stage, 18% (n = 20) had magnetic resonance imaging (MRI). For evaluation of metastases in patients with intermediate-risk disease, 24% (n = 8) had abdominopelvic computer tomography scan or MRI and bone scan. For evaluation of metastases in patients with high-risk disease, 9% (n = 6) had abdominopelvic computer tomography scan or MRI and bone scan. Concordance of tumor imaging to guidelines was poor overall (18%, n = 20), although it was significantly higher in Lagos (45%, n = 17) compared with Zaria (7%, n = 3) and Lafia (0%, n = 0; *P* < .001). More insured patients (36%) than uninsured patients (11%) had guideline-concordant imaging (*P* = .027).

**TABLE 3:** Imaging Used for Prostate Cancer Management and Its Concordance to Guidelines in Nigeria

Characteristic	Zaria, No. (%)	Lagos, No. (%)	Lafia, No. (%)	Total, No. (%)	*P* ^a^
	42 (38)	39 (35)	29 (26)	110 (100)	
Imaging used					
TRUSS					
No	12 (29)	24 (62)	29 (100)	65 (59)	<.001
Yes	30 (71)	15 (38)	0	45 (41)	
MRI					
No	39 (93)	22 (56)	29 (100)	90 (82)	<.001
Yes	3 (7)	17 (44)	0	20 (18)	
Skeletal X-ray					
No	26 (62)	33 (85)	21 (72)	80 (73)	.072
Yes	16 (38)	6 (15)	8 (28)	30 (27)	
AP USS					
No	12 (29)	24 (62)	3 (10)	39 (35)	<.001
Yes	30 (71)	15 (38)	26 (90)	71 (65)	
Bone scan					
No	41 (98)	27 (69)	29 (100)	97 (88)	<.001
Yes	1 (2)	12 (31)	0	13 (12)	
AP CT scan					
No	41 (98)	37 (95)	29 (100)	107 (97)	.432
Yes	1 (2)	2 (5)	0	3 (3)	
PET-CT scan					
No	42 (100)	39 (100)	29 (100)	110 (100)	NA
Yes	0	0	0	0	
Guideline concordance^b^					
Guideline-concordant cT imaging					
No	39 (93)	21 (55)	29 (100)	89 (82)	<.001
Yes	3 (7)	17 (45)	0	20 (18)	
Concordant metastasis imaging for low risk					
Not concordant	0	2 (40)	3 (100)	5 (56)	.126
Concordant	1 (100)	3 (60)	0	4 (44)	
Concordant metastasis imaging for intermediate risk					
Not concordant	2 (67)	8 (53)	16 (100)	26 (76)	.008
Concordant	1 (33)	7 (47)	0	8 (24)	
Concordant metastasis imaging for high risk					
Not concordant	38 (100)	12 (67)	10 (100)	60 (91)	<.001
Concordant	0	6 (33)	0	6 (9)	

Abbreviations: AP, abdominopelvic; CT, computed tomography; cT, clinical tumor; ISUP, International Society of Urological Pathology; MRI, magnetic resonance imaging; NA, not applicable; PET, positron emission tomography; TR, transrectal; USS, ultrasound.

^a^ *P* value from Pearson chi-squared test or Kruskal-Wallis test.

^b^ Guideline-concordant cT imaging = MRI; guideline-concordant metastasis imaging for low-risk = no additional imaging needed; guideline-concordant metastasis imaging for intermediate-risk (if ISUP grade ≥3) or high-risk = at least cross-sectional abdominopelvic imaging (CT or MRI) + bone scan.

**FIG 1:**
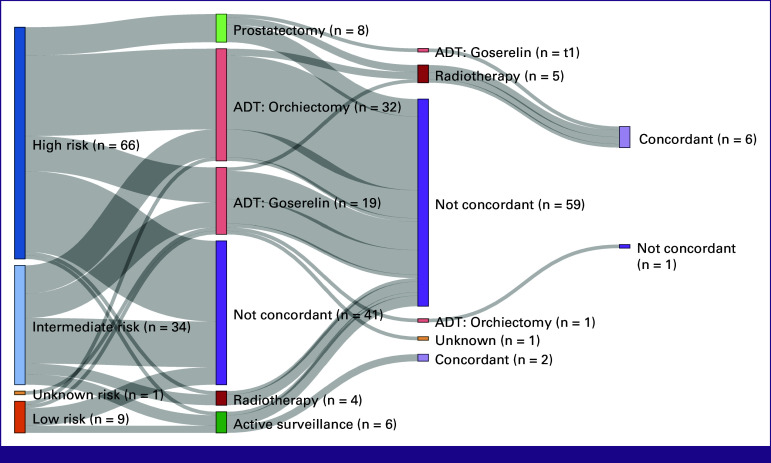
Sequence of treatment and concordance of management of low-risk, intermediate-risk, and high-risk groups of prostate cancer. ^a^See Appendix Table A3 for definitions of guideline-concordant treatment. ADT, androgen-deprivation therapy.

In the overall cohort, 8% (n = 9) had radical radiotherapy, 7% (n = 8) had radical prostatectomy, 47% (n = 52) had ADT, and 5% (n = 6) had active surveillance (Appendix Table A5). Among these patients, 5% (n = 6) had a combination of these treatments. Rates of guideline-concordant treatment were low overall (7%, n = 8), although they were significantly greater in Lagos (16%, n = 6) compared with Zaria (2%, n = 1) and Lafia (3%, n = 1; *P* = .046). In the three D'Amico risk groups, few patients also received treatment in concordance with guidelines with 22% (n = 2) in the low-risk, 0% (n = 0) in the intermediate-risk, and 9% (n = 6) in the high-risk groups (Fig 1; Table 2).

Retention in care after a median duration of 34 months was 43% (n = 47 of 109 patients), with 49% (n = 54 of 110 patients) having at least two follow-up visits within the first year of treatment. The Lagos site (*P* < .001), family history documentation (*P* = .011), and guideline-concordant imaging (*P* < .001) were significantly associated with retention in care (Appendix Table A6).

### Survival Characteristics

**FIG A1:**
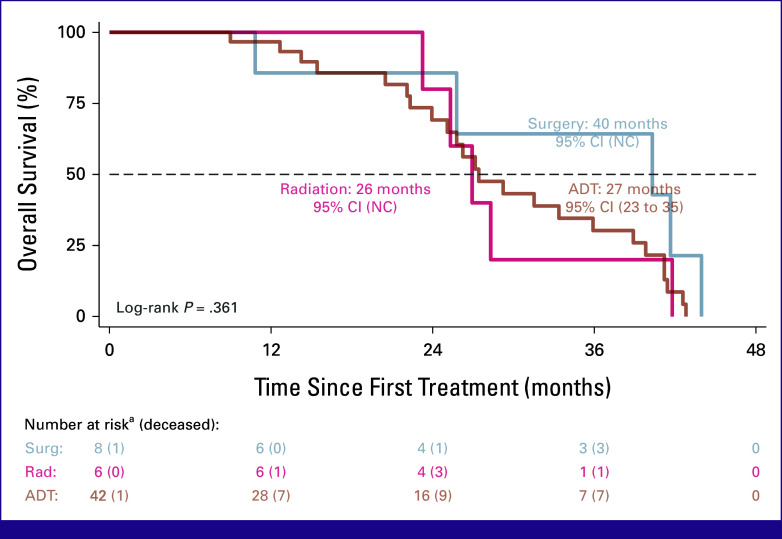
All-cause survival stratified by the first type of cancer-directed treatment received. ^a^A total of 42 ADT, eight RP, and six RT patients had observation data after treatment required for the Kaplan-Meier method. ADT, androgen-deprivation therapy; NC, not calculable because benchmark Kaplan-Meier estimate not met.

The 3-year all-cause survival was 41% (95% CI, 27 to 55) in the entire cohort. According to primary treatment received, 3-year all-cause survival was 64% (95% CI, 15 to 90) for radical surgery, 44% (95% CI, 24 to 63) for ADT alone, and 40% (95% CI, 5 to 75) for radical radiotherapy, with no significant difference across treatment groups (log-rank *P* = .361), as shown in Table 4 and Appendix Figure A1. Biochemical progression occurred in 18 of 54 patients with data, the 3-year recurrence-free survival was 59% (95% CI, 41 to 73), and median recurrence-free survival was 33 months. The stratification of survival by hospital site and disease characteristics is shown in Table 4. Delayed time to diagnosis beyond 30 days (log-rank test *P* = .770) and time to treatment beyond 90 days (log-rank test *P* = .089) did not have a statistically significant effect on recurrence-free survival (data not shown).

**TABLE 4:** All-Cause Survival, Recurrence-Free Survival, and Associations With Hospital Site and Disease Characteristics

Characteristic	Levels	All-Cause Survival				Recurrence-Free Survival			
		3-Year Survival, % (95% CI)	*P* ^a^	HR (95% CI)	*P* ^b^	3-Year Survival, % (95% CI)	*P* ^a^	HR (95% CI)	*P* ^b^
Hospital site	Zaria	NC	.066	1	Ref	NC	.109	1	Ref
	Lagos	49 (30 to 65)		0.58 (0.19 to 1.76)	.333	68 (48 to 82)		0.30 (0.09 to 0.97)	.045
	Lafia	33 (9 to 60)		1.35 (0.42 to 4.30)	.613	57 (22 to 81)		0.44 (0.11 to 1.79)	.252
Tumor stage	cT1	42 (15 to 67)	.635	1	Ref	66 (26 to 88)	.581	1	Ref
	cT2	51 (22 to 73)		0.69 (0.31 to 1.56)	.374	49 (20 to 74)		2.07 (0.51 to 8.31)	.307
	cT3	56 (20 to 80)		0.94 (0.38 to 2.31)	.889	61 (29 to 82)		1.54 (0.37 to 6.49)	.553
D'Amico risk	Low	25 (1 to 67)	.975	1		75 (13 to 96)	.069	1	
	Intermediate	37 (17 to 57)		0.83 (0.28 to 2.51)	.745	82 (44 to 95)		0.43 (0.04 to 4.79)	.496
	High (cT1-2)	40 (17 to 63)		0.77 (0.25 to 2.37)	.649	51 (26 to 72)		2.27 (0.29 to 18.0)	.437
	High (cT3)	60 (13 to 88)		0.82 (0.22 to 3.08)	.767	41 (10 to 71)		2.79 (0.32 to 24.0)	.350
Primary treatment	None	NC	.112	1	Ref	NC	.064	1	Ref
	Surgery	64 (15 to 90)		0.23 (0.03 to 1.58)	.133	21 (1 to 59)		1.33 (0.15 to 12)	.791
	Radiation	40 (5 to 75)		0.70 (0.22 to 2.18)	.537	48 (8 to 81)		0.46 (0.08 to 2.64)	.380
	ADT	44 (24 to 63)		0.53 (0.24 to 1.17)	.115	57 (35 to 75)		0.28 (0.09 to 0.89)	.032
	AS	60 (13 to 88)		0.37 (0.12 to 1.11)	.077	60 (13 to 88)		0.41 (0.16 to 1.03)	.057

Abbreviations: ADT, androgen-deprivation therapy; AS, active surveillance; cT, clinical tumor; HR, hazard ratio; NC, not calculable because benchmark Kaplan-Meier estimate not met; Ref, reference.

^a^ Log-rank test *P* value.

^b^ Wald test *P* value from Cox proportional hazards regression.

### Other Interhospital Disparities

A family history of prostate cancer was present in 25% (n = 13 of 52 patients with available data) and this was also significantly different across sites (*P* = .014). Table 5 shows how other clinical characteristics differ by hospital. Within the limitations of imaging evaluation for tumor and metastasis staging, more patients had low cT stage (cT1) in Lagos (48%, n = 10) compared with Lafia (14%, n = 4) and Zaria (n = 0; *P* < .001). Overall, 61% (n = 66) had D'Amico high-risk disease, and it was more likely (*P* < .001) in Zaria (90%, n = 38) compared with Lagos (47%, n = 18) or Lafia (34%, n = 10).

**TABLE 5:** Clinical Characteristics of Prostate Cancer Management in Nigeria

Characteristic^a^	Zaria, No. (%)	Lagos, No. (%)	Lafia, No. (%)	Total, No. (%)	*P* ^b^
	42 (38)	39 (35)	29 (26)	110 (100)	
Family history					
No	1 (100)	18 (60)	20 (95)	39 (75)	.014
Yes	0	12 (40)	1 (5)	13 (25)	
Missing data	41	9	8	58	
Comorbid conditions					
No	17 (41)	17 (55)	7 (25)	41 (41)	.067
Yes	24 (59)	14 (45)	21 (75)	59 (59)	
Missing data	1	8	1	10	
PSA at diagnosis, ng/mL					
<10	15 (36)	14 (36)	16 (59)	45 (42)	.089
10-20	6 (14)	11 (28)	5 (19)	22 (20)	
>20	21 (50)	14 (36)	6 (22)	41 (38)	
Missing data	0	0	2	2	
cT stage					
cT1	0	10 (48)	4 (14)	14 (16)	<.001
cT2	22 (61)	8 (38)	13 (45)	43 (50)	
cT3	14 (39)	3 (14)	12 (41)	29 (34)	
Not documented	6	18	0	24	
Gleason's score					
2-6	16 (39)	6 (15)	4 (15)	26 (24)	.162
7 (3 + 4)	7 (17)	10 (26)	7 (26)	24 (22)	
7 (4 + 3)	6 (15)	10 (26)	6 (22)	22 (21)	
8	6 (15)	6 (15)	9 (33)	21 (20)	
9	4 (10)	5 (13)	1 (4)	10 (9)	
10	2 (5)	2 (5)	0	4 (4)	
Missing data	1	0	2	3	
D'Amico risk group at diagnosis					
High	38 (90)	18 (47)	10 (34)	66 (61)	<.001
Intermediate	3 (7)	15 (39)	16 (55)	34 (31)	
Low	1 (2)	5 (13)	3 (10)	9 (8)	
Missing data	0	1	0	1	

Abbreviations: cT, clinical tumor; PSA, prostate-specific antigen.

^a^ Percentages and association tests were computed for variables with known values.

^b^ *P* value from Pearson chi-squared test or Kruskal-Wallis test.

## DISCUSSION

Clinically localized prostate cancer represents an opportunity to cure patients and improve survival. However, our results demonstrate delays in treatment initiation; suboptimal quality of staging, treatment, and care process (<70% benchmark); poor patient survival outcomes; and geographic disparities across three sites in Nigeria.

With respect to the time to treatment received by patients with prostate cancer in Nigeria, our finding of a median time of 68 days is longer than the minimum standard for cancer treatment in Brazil (≤60 days) and the recommendations of the European Union (≤15 working days), United Kingdom (≤31 days), and Columbia (<30 days).^15^ Considering the specific treatment received, the median time to ADT of 68 days was within the United States standard for cytotoxic chemotherapy of <120 days.^15^ This could suggest that access to ADT was good. As the mechanism of action and adverse effects of ADT are different from those of cytotoxic chemotherapy, this could also imply a need for a consensus definition of a benchmark regarding the optimal timing for commencing ADT treatment. However, the medium time to surgery (104 days) was longer than the target time in Australia (≤35 days) and the medium time to radiotherapy (117 days) was longer than the target time in Canada (<28 days).^15^ This treatment delay could imply that the use of more complex treatment options added another layer of barrier to accessing care because of the increased need for infrastructure, manpower, and social and financial resources.^16,17^

Similar to our study where only 7% had a treatment wait time of 30 days and below, Omisanjo et al^18^ in Nigeria also reported that 6.1% of their patients had a waiting time of <28 days between presentation and treatment. These findings mean that the initiation of the first treatment, especially in relation to radical prostatectomy and radical radiotherapy, is longer than most international standards. This study used established quality metrics from a systematic review by Gori et al,^13^ from the RAND Corporation, and from the ASCO Quality Oncology Practice Initiative to assess quality.^11,12^ Although this makes the result not just comparable with figures from other countries but also valuable in developing actionable interventions, it is plausible that big differences in time to treatment standards between Nigeria and high-income countries are possible contributors to the outcome disparities observed. The variation in the recommended treatment interval between countries, sites of cancer, and types of treatment, therefore, underlies the need for the adaptation and validation of quality indicators to evaluate the management of prostate cancer within the context of Nigeria and sub-Saharan Africa.

The finding that a low-risk Gleason's score (2-6) is associated with double the time to start treatment might mean that treatment decision making between ADT versus active surveillance and radical treatment is complex for patients. It could also imply that it is perceived to be less urgent by doctors because of prioritization of patients with high-risk disease amid limitations in manpower for radical prostatectomy and a low number of radiotherapy facilities.

The median time from tertiary hospital presentation to diagnosis of 31 days in this study is close to the ≤28-day standard for cancer diagnostic interval in the United Kingdom.^15^ Osowiecka et al^19^ in Poland and Singh et al^20^ in South Africa reported longer time to diagnosis of 7.7 weeks (54 days) and 100 days. However, their defined intervals began with time from cancer suspicion and time from referral from peripheral health facilities, respectively, rather than presentation to the urologist in the tertiary hospital setting. For our study, data related to patients' presentation in peripheral health facilities before referral to a tertiary hospital were not available. Our previous qualitative research experience suggests that the prespecialist referral process is delayed by difficulties such as poor provider knowledge, widespread negative beliefs, and the absence of referral linkages at the primary and secondary health care levels.^21^ It is known that the decentralization of services for communicable diseases such as retroviral disease and noncommunicable diseases such as hypertension has improved access to care. The finding of a high rate of medical comorbidities (59%) in this patient cohort highlights the overlap of other noncommunicable diseases with that of prostate cancer. It is therefore possible that patients who are already in the system seeking care for another noncommunicable disease are more likely to be diagnosed with prostate cancer while still at the early stages whereas patients with no comorbidities might be out of medical care and present at later stages. This highlights the opportunity to explore the integration of services in the nonspecialist setting and the use of novel technologies to enhance pathologic reporting to optimize the early detection of prostate cancer and improve care quality in Nigeria.^22^ Looking at the postdiagnosis period, 39% of patients received no treatment after a biopsy-confirmed prostate cancer diagnosis in our patient cohort. This could mean that these patients had various barriers in accessing appropriate cancer management services after a biopsy confirmation, which represents a key target for navigation intervention to improve timely and quality care.

Regarding the quality of staging imaging, our study reported an overall poor rate of guideline-concordant imaging, with significant differences by patient age, location, and insurance status, and a suggestion of differences by D'Amico risk score. Observations by Makarov et al^23^ noted discordance of prostate cancer imaging with guidelines in the United States. The low rate of guideline-concordant imaging is likely because of out-of-pocket costs that are prohibitive for many patients. It could also be due to the nonavailability or poor functionality of imaging facilities at the locations and as a result of the nonuniformity in guidelines used by specialists.^21^ Stakeholders in our qualitative study proposed that guideline concordance could be improved by a local domestication that aligns with the needs and affordability of patients and the resources available in the health care system.^21^ Region-specific protocols such as the National Comprehensive Cancer Network resource-stratified guideline for sub-Saharan Africa is also a valuable tool for adoption.^21^ To balance the goal of improved care quality with the challenge of equitable access to treatment, tailored interventions to improve guideline concordance should be implemented and evaluated.

With respect to treatment quality, a majority of the patients in this study did not receive curative treatment. This could reflect limitation in the use of nonconcordant staging investigations for the selection of radical treatment. As patients in this cohort presented in the seventh decade of life in consonance with other Nigerian studies,^24,25^ and had medical comorbidities, the result of noncurative treatment could also be due to the possible provider perception of poor life expectancy in the elderly age group despite presumably early disease. This, therefore, points to the need for formal geriatric evaluation in the elderly population to objectively assess and better understand their overall health and prostate cancer status.

Looking at the quality of the care process, there was a high rate of loss to follow-up (58%) in this Nigerian cohort, with only 42% retained in care after a median duration of 34 months. This value is higher than the loss to follow-up rate in Benin Republic (49%) and Cote d'Ivoire (36%).^26^ Along with the suboptimal performance on quality metrics at some sites, the high rate of loss to follow-up highlights challenges to repeated hospital visits during the cancer management journey of patients and indicates the poor quality of care received by patients with prostate cancer in Nigeria.^21^ These findings thus re-emphasize suggestions for greater insurance coverage, better provider-to-patient communication, and structured navigation and social support systems to surmount shortcomings during patient management in Nigeria.^21^ The Lancet Global Health Commission reports the relatively higher significance of low-quality care in predicting the occurrence of cancer deaths. There is therefore a need for the implementation of quality improvement programs and their ongoing evaluation to strengthen the standard of care in Nigeria.^22^

The 3-year all-cause survival rate of 41% in this study is similar to the reported rate of 49% in some countries in sub-Saharan Africa,^26^ but poor in relation to the all-cause survival rate in the United States (over 90%).^27^ This could be explained by possible understaging and the use of suboptimal treatment in our patient cohort.^5^ It could also be due to the relatively lower life expectancy in Nigeria in comparison with high-income countries. Our result that time to diagnosis within 30 days and treatment within 90 days does not affect recurrence-free survival could be reassuring to patients who might experience these delays. Gupta et al^28^ noted similar results for treatment waiting time of up to 6 months in those undergoing radical prostatectomy. Conversely, real-world evidence on improved outcomes after concordant timing of care is limited.^22^ Therefore, more studies are needed on the impact of time to care on survival. Also, addressing diagnostic and treatment delays is a matter of equity so that all patients can receive comparable quality of care.^15^

There were significant disparities in the socioeconomic status, clinical characteristics, and treatment quality across the three study sites in Nigeria. A higher percentage had low or retired occupation status, and a higher rate had a lack of health insurance in Zaria and Lafia compared with Lagos. In particular, a greater proportion of patients from Zaria had high-risk disease. However, turning to treatment quality, patients in Lagos had greater access to MRI and bone scans, and received imaging and treatment that had greater concordance to guidelines. This disaggregation uncovers previously unknown inequity in prostate cancer patient characteristics and treatment quality, and supports our perception of neighborhood deprivation for patients in the northern zones of Nigeria. Examining these findings within the lens of the observation by Hamdi et al^29^ of an inverse relationship between the human development index and cancer outcomes in Africa, the northern zones of Nigeria represent a unique population with wider social and clinical need that requires targeted intervention. These findings underscore a great need to categorize health and access data in Nigeria by geographical location rather than by complex multiethnic identifiers in a country with over 225 million people, 300 ethnic groups, and 500 languages.

Our study limitations include a sample population that is only representative of patients receiving health care in the public tertiary hospital setting in Nigeria, with information on care outside the specialist hospital not available. In addition, as a retrospective study, we were limited by the quality and availability of clinical data, with several variables affected by missing data. We hope to broaden our collaboration with health care providers during the future development and implementation of targeted interventions to enhance the applicability of the study results.

In conclusion, this study highlights important markers of quality and equity characterizing early-stage prostate cancer diagnosis, staging, and treatment in Nigeria. This study demonstrated delayed time to treatment; suboptimal quality of staging, treatment, and care process; poor patient survival; and geographic disparities. These gaps should be the target for interventions to expand access to health care and improve care delivery.

## APPENDIX

**TABLE A1:** Background of the Study Centers

Available Resources^a^	Zaria	Lagos	Lafia^b^
Bed capacity	730	950	450
Multidisciplinary specialists			
Chemical pathologist	6	4	1
Histopathologist	13	8	1
Oncologist	5	10	1
Oncology nurses	6	10	1
Radiologist	11	6	3
Urologist	8	5	3
Advanced imaging facilities			
CT scan	1	2	1
MRI	2	1	0
Bone scan^c^	0	0	0
PET-CT scan	0	0	0
Radiotherapy facilities			
Brachytherapy	1	3	0
Teletherapy	1	1	0

Abbreviations: CT, computed tomography; MRI, magnetic resonance imaging; PET, positron emission tomography; PSA, prostate-specific antigen.

^a^ Patients are referred from peripheral health care centers, family physicians, and other specialists after suspicion of prostate cancer on account of difficulty in urination, abnormal prostate on digital rectal examination, or elevated PSA. These patients then present to the urology outpatient clinic at the tertiary hospital for confirmatory diagnosis. In the absence of complete blood work, the urologist orders PSA tests. After evaluation, the urologist schedules a core biopsy of the prostate in the presence of prostate biopsy indications. If biopsy is positive, the urologist further orders imaging to clinically stage the prostate cancer. If tumor is clinically localized, radical prostatectomy, radical radiotherapy, active surveillance, and androgen-deprivation therapy are considered as options of treatment. Payment for these services is predominantly through a fee-for-service model.

^b^ Nearest MRI and radiotherapy facilities are within a distance of 100 km and 180 km, respectively, from the Lafia study center.

^c^ Nearest bone scan facilities are within a distance of 300 km, 10 km, and 180 km from the Zaria, Lagos, and Lafia study centers, respectively.

**TABLE A2:** Definition of Data Variables and Study End Points

Data Categories	Variables	Study End Points and Analysis^a^^,^^b^
Hospital site	Zaria/Lafia/Lagos	Interhospital difference in end points
Demographics	Age at diagnosis	Sociodemographic characteristics
	Marital status	
Socioeconomic status	Occupation	
	Education	
	Health insurance	
	Receiving care for another condition at the hospital	
Clinical characteristics	Family history	Clinical characteristics
	Presence of comorbid conditions	
	PSA at diagnosis	
	cT stage	
	Gleason's score	
	Calculated D'Amico risk group, which includes Gleason's score, PSA, and cT stage^c^	
Quality metrics	Imaging used: TRUSS/MRI/X-ray/AP USS/bone scan/AP CT scan/PET-CT scan	Guideline-concordant staging investigations^c^
	Definitive treatment received: radical prostatectomy/radical radiotherapy/androgen deprivation therapy (goserelin/orchiectomy)/active surveillance	Guideline-concordant treatment^c^
	Dates of First cancer-related hospital presentation Prostate biopsy Initiation of each treatment	Time to treatment^d^ Time to diagnosis^d^
	Documentation of Family history PSA level Number of tissue cores taken at the time of prostate biopsy Gleason's grade	Other established prostate cancer quality metrics^e^—the proportion of patients with An assessment of family history of prostate cancer A minimum of 10 cores taken at the time of prostate biopsy Documentation of Gleason's grade cT staging documented within 1 month of diagnosis At least two follow-up hospital visits during the first year after treatment
	Timing of cT stage documentation Number and date(s) of follow-up appointment(s) Retention in care at the end of follow-up	
Patient survival	Dates of first biochemical recurrence Vital status (alive/dead) Date of death	Biochemical recurrence-free survival All-cause survival

Abbreviations: AP, abdominopelvic; CT, computed tomography; cT, clinical tumor; MRI, magnetic resonance imaging; PET, positron emission tomography; PSA, prostate-specific antigen; TR, transrectal; USS, ultrasound.

^a^ The primary end point was time to treatment. All other end points are secondary end points.

^b^ Sociodemographic and clinical characteristics, imaging used, definitive treatment received, and quality metrics and their stratification by hospital site were computed using descriptive statistics. The association of hospital site, sociodemographic and clinical characteristics, and quality metrics to the time to treatment and the time to diagnosis was evaluated using Cox regression analysis. Interhospital differences in the sociodemographic and clinical characteristics, quality metrics, and imaging used were analyzed using Pearson chi-squared test or Kruskal-Wallis test. Recurrence-free survival and all-cause survival were computed using Kaplan-Meier technique and Cox regression. The effect of time to diagnosis and time to treatment on recurrence-free survival was further evaluated with a log-rank test and hazard ratio.

^c^ Concordance to the standard of care was analyzed using the European Association of Urology guidelines.^14^ See definitions of the risk groups and guideline concordance in Appendix Table A3.

^d^ Time to treatment = the time interval from the date of prostate biopsy to the date of initiation of first cancer-directed treatment or of active surveillance; time to diagnosis = time interval between the date of first cancer-related presentation at a tertiary hospital to the date of prostate biopsy.

^e^ Other established quality metrics were extracted from a systematic review by Gori et al,^13^ from the RAND Corporation, and from the ASCO Quality Oncology Practice Initiative.^11,12^

**TABLE A3:** Definition of D'Amico Risk and Concordance to Standard of Imaging and Treatment Using the European Association of Urology Guidelines as a Benchmark

Stage	Risk Category	Definition
Guideline-concordant imaging		
Localized	Low risk Gleason's score ≤6 and PSA <10 ng/mL and Clinical stage T1-T2a	cT staging: MRI Metastasis staging: no additional imaging
Localized	Intermediate risk Gleason's score = 7 or PSA 10-20 ng/mL or Clinical stage = T2b	cT staging: MRI Metastasis staging (if ISUP grade ≥3): at least cross-sectional abdominopelvic imaging (CT or MRI) + bone scan
Localized	High risk Gleason's score ≥ T2c or PSA >20 ng/mL or Gleason's score ≥8	cT staging: MRI Metastasis staging: at least cross-sectional abdominopelvic imaging (CT or MRI) + bone scan
Locally advanced		cT staging: MRI Metastasis staging: at least cross-sectional abdominopelvic imaging (CT or MRI) + bone scan
Guideline-concordant treatment		
Localized	Low risk Gleason's score ≤6 and PSA <10 ng/mL and Clinical stage T1-T2a	Active surveillance (assumption is that all have life expectancy >10 years)
Localized	Intermediate risk Gleason's score = 7 or PSA 10-20 ng/mL or Clinical stage = T2b	Radical prostatectomy Radical radiotherapy + short-term ADT
Localized	High risk Gleason's score ≥ T2c or PSA >20 ng/mL or Gleason's score ≥8	Radical prostatectomy + (radical radiotherapy or ADT) Radical radiotherapy + long-term ADT
Locally advanced		Radical prostatectomy + (radical radiotherapy or ADT) Radical radiotherapy + ADT ADT monotherapy if PSA doubling time <12 months plus (PSA >50 ng/mL or grade group 5 or troublesome local disease-related symptom)

NOTE. ADT in the context of definitive treatment means the use of orchiectomy or luteinizing hormone–releasing hormone analogs, for example, goserelin and leuprolide.

Abbreviations: ADT, androgen-deprivation therapy; CT, computerized tomography; cT, clinical tumor; ISUP, International Society of Urological Pathology; MRI, magnetic resonance imaging; PSA, prostate-specific antigen; T, tumor.

**TABLE A4:** Time to Treatment According to cT Stage and D'Amico Risk Group

Characteristic	Any Treatment	Radical Prostatectomy	Radical Radiotherapy	ADT	Active Surveillance
cT stage					
cT1	74 (60-134)	NA	117 (80-152)	97 (69-394)	60 (42-63)
cT2	74 (50-120)	104 (0-134)	NA	70 (53-113)	53 (50-57)
cT3	67 (51-221)	NA	NA	67 (51-221)	NA
Risk groups					
Low risk	222 (42-391)	NA	NA	391 (388-394)	42 (28-57)
Intermediate risk	75 (64-117)	NA	117 (80-152)	75 (66-190)	63 (57-64)
High risk	64 (49-109)	104 (0-134)	NA	60 (49-99)	50 (50-50)

NOTE. All time to treatment are expressed in days.

Abbreviations: ADT, androgen-deprivation therapy; cT, clinical tumor; NA, not applicable.

**TABLE A5:** Type of Treatment According to D'Amico Low-Risk, Intermediate-Risk, and High-Risk Groups of Prostate Cancer

Treatment Types	Total, No. (%)	D'Amico Risk Groups, No. (%)		
		High	Intermediate	Low
ADT: orchiectomy	33 (100)	24 (73)	8 (24)	1 (3)
ADT: goserelin	19 (100)	11 (58)	7 (37)	1 (5)
Radical radiotherapy	9 (100)	6 (67)	3 (33)	0
Radical prostatectomy	8 (100)	8 (100)	0	0
Active surveillance	6 (100)	1 (17)	3 (50)	2 (33)
No treatment^a^	43 (100)	25 (58)	13 (30)	5 (12)

Abbreviations: ADT, androgen-deprivation therapy; cT, clinical tumor.

^a^ Among patients who did not receive any treatment, two (5%), 16 (37%), and 13 (30%) had cT stage I, II, and III, respectively, while cT was unknown in 12 (28%).

**TABLE A6:** Retention in Care

Characteristic	Retained	Lost to Follow-Up	Total	*P* ^a^
	47 (43)	62 (57)	109 (100)	
Hospital site, No. (%)				
Zaria	4 (9)	37 (60)	41 (38)	<.001
Lagos	32 (68)	7 (11)	39 (36)	
Lafia	11 (23)	18 (29)	29 (27)	
Education, N (%)				
Nonformal to secondary	10 (29)	13 (42)	23 (35)	.255
Tertiary	25 (71)	18 (58)	43 (65)	
Occupation class, No. (%)				
Class II-III or none	30 (79)	46 (82)	76 (81)	.699
Class I (high)	8 (21)	10 (18)	18 (19)	
Insurance status, No. (%)				
No	27 (82)	52 (91)	79 (88)	.189
Yes	6 (18)	5 (9)	11 (12)	
Receiving care for another condition, No. (%)				
No	25 (68)	36 (63)	61 (65)	.662
Yes	12 (32)	21 (37)	33 (35)	
Documentation of family history, No. (%)				
No	18 (38)	39 (63)	57 (52)	.011
Yes	29 (62)	23 (37)	52 (48)	
Documentation of cT stage within 1 month, No. (%)				
No	32 (68)	32 (52)	64 (59)	.084
Yes	15 (32)	30 (48)	45 (41)	
Guideline-concordant metastasis imaging, No. (%)				
Not concordant	29 (63)	61 (98)	90 (83)	<.001
Concordant	17 (37)	1 (2)	18 (17)	
Guideline-concordant treatment, No. (%)				
Not concordant	40 (87)	60 (97)	100 (93)	.054
Concordant	6 (13)	2 (3)	8 (7)	
Days to diagnosis				
Median (IQR)	34 (20-50)	28 (10-50)	31 (13-50)	.222
Count of patients, No.	46	56	102	
Days to treatment				
Median (IQR)	69 (55-117)	64 (46-178)	68 (51-120)	.589
Count of patients, No.	39	28	67	
PSA				
Median (IQR), ng/mL	13 (1-34)	13 (1-41)	13 (1-39)	.935
Count of patients, No.	47	60	107	

NOTE. Percentages and association tests were computed for variables with known values.

Abbreviations: cT, clinical tumor; PSA, prostate-specific antigen.

^a^ *P* value from Pearson chi-squared test.
